# Removing the FDA’s Boxed Hepatotoxicity Warning and Liver Function Testing Requirement for Ambrisentan

**DOI:** 10.1001/jamanetworkopen.2024.19873

**Published:** 2024-07-18

**Authors:** William B. Feldman, Mufaddal Mahesri, Ameet Sarpatwari, Krista F. Huybrechts, Yanmin Zhu, Catherine S. Hwang, Joyce Lii, Su Been Lee, Sushama Kattinakere Sreedhara, Gita A. Toyserkani, Esther H. Zhou, Laura Zendel, Cynthia LaCivita, Claudia Manzo, Gerald J. Dal Pan, Aaron S. Kesselheim, Katsiaryna Bykov

**Affiliations:** 1 Division of Pharmacoepidemiology and Pharmacoeconomics, Department of Medicine, Brigham and Women’s Hospital and Harvard Medical School, Boston, Massachusetts; 2 Program on Regulation, Therapeutics, and Law, Division of Pharmacoepidemiology and Pharmacoeconomics, Department of Medicine, Brigham and Women’s Hospital and Harvard Medical School, Boston, Massachusetts; 3 Division of Pulmonary and Critical Care Medicine, Department of Medicine, Brigham and Women’s Hospital and Harvard Medical School, Boston, Massachusetts; 4 Office of Surveillance and Epidemiology, Center for Drug Evaluation and Research, Food and Drug Administration, Silver Spring, Maryland

## Abstract

**Importance:**

Endothelin receptor antagonists are first-line therapy for pulmonary arterial hypertension (PAH). The first 2 agents approved in the class, bosentan and ambrisentan, initially carried boxed warnings for hepatotoxicity and required monthly liver function tests (LFTs) as part of a risk evaluation and mitigation strategy (REMS); however, in 2011, as further safety data emerged on ambrisentan, the boxed hepatotoxicity warning and LFT requirements were removed.

**Objective:**

To analyze changes in the use of and LFT monitoring for ambrisentan and bosentan after changes to the ambrisentan labeling and REMS.

**Design, Setting, and Participants:**

This serial cross-sectional study used data from 3 longitudinal health care insurance claims databases—Medicaid, Optum’s deidentified Clinformatics Data Mart, and Merative Marketscan—to perform an interrupted time series analysis of prescription fills and LFTs for patients taking ambrisentan and bosentan. Participants were patients filling prescriptions for ambrisentan and bosentan from July 1, 2007, to December 31, 2018. Data analysis was performed from April 2021 to August 2023.

**Exposure:**

Removal of the boxed warning for hepatotoxicity and the REMS LFT monitoring requirements on ambrisentan in March 2011.

**Main Outcomes and Measures:**

The primary outcomes were use of ambrisentan (ie, individuals with at least 1 dispensing per 1 000 000 individuals enrolled in the 3 datasets) vs bosentan and LFT monitoring (ie, proportion of initiators with at least 1 ordered test) before initiation and before the first refill.

**Results:**

A total of 10 261 patients received a prescription for ambrisentan during the study period (7442 women [72.5%]; mean [SD] age, 52.6 [17.6] years), and 11 159 patients received a prescription for bosentan (7931 women [71.1%]; mean [SD] age, 47.7 [23.7] years). Removal of the ambrisentan boxed hepatotoxicity warning and LFT monitoring requirement was associated with an immediate increase in the use of ambrisentan (1.50 patients per million enrollees; 95% CI, 1.08 to 1.92 patients per million enrollees) but no significant change in the use of bosentan. There were reductions in recorded LFTs before drug initiation (13.1% absolute decrease; 95% CI, −18.2% to −8.0%) and before the first refill (26.4% absolute decrease; 95% CI, −34.4% to −18.5%) of ambrisentan but not bosentan.

**Conclusions and Relevance:**

In this serial cross-sectional study of ambrisentan, labeling changes and removal of the REMS-related LFT requirement were associated with shifts in prescribing and testing behavior for ambrisentan but not bosentan. Further clinician education may be needed to maximize the benefits of REMS programs and labeling warnings designed to ensure the safe administration of high-risk medications.

## Key Points

**Question:**

How did use of and liver function tests (LFTs) for ambrisentan vs bosentan change after the boxed hepatotoxicity warning and LFT requirement under the risk evaluation and mitigation strategy (REMS) were removed for ambrisentan but not bosentan?

**Findings:**

In this serial cross-sectional study using interrupted time series analysis, these changes were associated with an immediate increase in the use of ambrisentan (1.50 patients per million enrollees) but no significant change in the use of bosentan. There were statistically significant reductions in recorded LFTs before drug initiation (13.1% absolute decrease) and before first refill (26.4% absolute decrease) of ambrisentan but not bosentan.

**Meaning:**

These findings suggest that labeling changes and removal of the REMS-related LFT requirement were associated with shifts in prescribing and testing behavior for patients taking ambrisentan and that further clinician education may be needed to ensure the safe administration of high-risk medications.

## Teaser

This cross-sectional study examines changes in the use of and liver function test monitoring for ambrisentan and bosentan after changes by the US Food and Drug Administration to the ambrisentan labeling and risk evaluation and mitigation strategy.

## Introduction

Endothelin receptor antagonists are first-line therapy for the management of pulmonary arterial hypertension (PAH).^1^ PAH is characterized by elevated blood pressure in the pulmonary vasculature that can lead to shortness of breath and increased mortality. Endothelin receptor antagonists blunt the effects of endothelin, a potent vasoconstrictor released by vascular endothelial cells,^2^ and reduce pulmonary arterial pressures, leading to increased exercise capacity and improved functional status for those with PAH.^3^ Endothelin was first discovered in 1988,^2^ and the first endothelin receptor antagonist to receive US Food and Drug Administration (FDA) approval was bosentan (Tracleer; Johnson & Johnson) in 2001.^4^ However, the 2 pivotal clinical trials leading to FDA approval also found a substantial number of patients who had elevated liver function enzymes,^5,6^ prompting a large postmarketing surveillance study in Europe that found a 10.1% annual rate of liver enzyme abnormalities in 4623 patients treated with bosentan.^7^ Although the precise mechanism of liver injury is unknown (impairment of the bile salt transporter pump has been partially implicated), elevated risks of hepatotoxicity have consistently been observed in clinical trials and observational research among those taking bosentan.^3,8^

Given these risks, the FDA approved bosentan with a boxed warning for hepatotoxicity, and the manufacturer voluntarily implemented a safety program to ensure proper monitoring of liver function tests (LFTs), including alanine aminotransferase, aspartate aminotransferase, and bilirubin. The program instructed prescribers to order LFTs before initiating therapy and then monthly thereafter while a patient is receiving therapy to detect early signs of liver injury and adjust or discontinue the medication as needed. In 2007, the FDA gained the authority to require such safety programs for certain drugs with serious risks to help ensure that their benefits outweigh the risks.^9,10,11^ These risk evaluation and mitigation strategies (REMS) may involve patient monitoring, dispensing limits, educational interventions, and other activities designed to minimize the risks of a drug.^9^ For bosentan, the precursor safety program was deemed by the FDA to be a REMS.^12^

By the time the FDA approved the second endothelin receptor antagonist, ambrisentan (Letairis; Gilead), in 2007, case reports of liver enzyme abnormalities had already emerged for another endothelin receptor antagonist, sitaxentan (Thelin; Pfizer), which was sold in Europe and was later withdrawn because of cases of fatal liver injury (the drug was never approved in the US).^8^ Despite ambrisentan being a more selective endothelin receptor antagonist than bosentan or sitaxentan,^3^ the FDA required a boxed warning for hepatotoxicity on ambrisentan given the risk of class effects, and a similar safety program before becoming a REMS in 2007.^13^ However, in the years following, further safety data emerged showing that ambrisentan lacked the major hepatotoxic effects of bosentan. In March 2011, the manufacturer and FDA agreed to remove ambrisentan’s boxed warning for hepatotoxicity and the LFT monitoring component of the REMS, although both remained in place for bosentan.^14^

We performed an interrupted time series analysis to examine how removal of the boxed warning for hepatotoxicity and associated REMS requirements on ambrisentan was associated with prescribing patterns and LFT monitoring. We hypothesized that use of ambrisentan would increase after removal of the hepatotoxicity boxed warning and REMS requirement while LFT monitoring would decrease, but that similar changes would not be observed among patients prescribed bosentan.

## Methods

### Data Source

This serial cross-sectional study used data from 3 large national health care insurance claims databases: (1) Medicaid, the joint state-federal public health insurance program in the US for individuals with lower income; (2) Optum’s deidentified Clinformatics Data Mart Database, which is derived from a database of administrative health claims for members of large commercial and Medicare Advantage health plans; and (3) Merative Marketscan, a large commercial health insurance database. All 3 databases contain patient-level information for inpatient and outpatient services with accompanying diagnosis and procedure codes, filled outpatient prescriptions, and demographic information for all enrollees. The Medicaid database has data from all 50 states and the District of Columbia, and the 2 commercial databases also cover large, geographically diverse populations and primarily include patients with private insurance.^15^ This study was approved by the Mass General Brigham and FDA institutional review boards. Because it was conducted using deidentified data, informed consent was not needed, in accordance with 45 CFR §46. The study followed the Strengthening the Reporting of Observational Studies in Epidemiology (STROBE) reporting guidelines for serial cross-sectional studies.^16^

### Study Population and Study Design

The study population included individuals with at least 1 dispensing of ambrisentan or bosentan between July 1, 2007, and December 31, 2018. The study period started during the month of ambrisentan approval (July 2007) and ended on the last month when data were available from all 3 databases (December 2018). We excluded Medicaid enrollees aged 65 years and older because they qualified for Medicare and might, therefore, have had incomplete claims. We required eligible Medicaid beneficiaries to be enrolled in fee-for-service plans or managed care plans with evidence of complete claims and dispensing data. Ambrisentan and bosentan dispensing were identified using pharmacy claims that included dates of dispensing and National Drug Codes linked to drug names. Because the boxed warning for hepatotoxicity removal and REMS change for ambrisentan occurred in March 2011, we compared trends after March 2011 with trends before March 2011 using segmented regression analysis for interrupted time series.^17^

### Outcomes

We measured 2 outcomes in the study: drug use and LFT monitoring. Drug use was assessed for each month of the study period and was defined as the number of enrollees with at least 1 ambrisentan dispensing (regardless of the prescription duration or whether the prescription was for an initial fill or refill) per million individuals who were enrolled in their health insurance plan for at least 28 days that month.

The occurrence of LFTs was assessed in individuals who initiated ambrisentan therapy and was defined as the proportion of initiators who underwent LFTs. Initiators were defined as patients with at least 6 months of insurance enrollment before ambrisentan initiation and no evidence of ambrisentan use in those 6 months. Test orders were identified using the *Current Procedural Terminology* and *Healthcare Common Procedure Coding System* codes 84460, 80076, 80054, 80058, 80053 or 84450 (eTable 1 in Supplement 1). Because tests ordered in a hospital setting would not be billed separately to insurance, we assumed that tests were done for patients who were hospitalized. Thus, a hospitalization during the assessment period counted as evidence of LFTs in our analyses. Because REMS requirements included baseline tests before treatment initiation and monthly tests during follow-up, we used claims data to evaluate (1) whether an LFT was completed within 90 days before and including the date of drug initiation, and (2) whether an LFT was completed within 31 days before and including the date of the first refill. The latter measure was analyzed for initiators who had at least 1 refill following initiation of the drug. As control outcomes, we evaluated use and LFT monitoring among bosentan initiators. With the exception of the National Drug Codes used to identify drug fills, all definitions for drug initiation and LFT monitoring were repeated in the analyses of bosentan.

### Statistical Analysis

Drug use was plotted for each month of the study period. In the interrupted time series analysis, we compared monthly use of ambrisentan and bosentan during the 2 years preceding removal of the boxed warning for hepatotoxicity and REMS LFT requirement (March 2009 to February 2011) and the 2 years following (April 2011 to March 2013), excluding the month of change (March 2011). The analysis was limited to 2 years before and 2 years after the labeling and REMS modification to ensure that the trends were not affected by other changes that occurred during the study period, such as the approval of other medications for PAH. The changes in trends during the baseline and postintervention periods were assessed using ordinary least-squares regression by fitting a segmented linear regression model with Newey-West SEs and a 1-month lag (March 2011). We also performed a sensitivity analysis with a lag of 3 months (March 2011 to May 2011) and compared trends from March 2009 to February 2011 (baseline period) to trends from June 2011 to March 2013 (postintervention period). All analyses were weighted by the number of eligible enrollees per month.

LFT monitoring was also plotted over time but in 6-month rather than 1-month increments owing to the small number of initiators. For each 6-month period starting in July 2007, we plotted the proportion of initiators who (1) had LFTs completed during the baseline period (90 days leading up to and including the date of the initial dispensing), and (2) had LFTs completed before the first refill (31 days leading up to and including the date of the first refill). Median (IQR) percentages were calculated for the preintervention (July 2007 to December 2010) and postintervention (July 2011 to December 2018) periods. Because of the small number of data points, we used all available data for the analysis of trends and started the analysis in July 2007 (unlike the analyses of monthly use, for which we used the 2 years leading up to and following the REMS change). The baseline period for our analysis of LFTs was, therefore, from July 2007 to December 2010, and the postintervention period was from July 2011 to December 2018 (with the period of the REMS change, January 2011 to June 2011, excluded). Testing before drug initiation and during therapy were separately evaluated. All time series analyses were weighted by the number of initiators during each 6-month period and were conducted using ordinary least-squares regression with Newey-West SEs. Data analysis was performed from April 2021 to August 2023. Statistical analyses were performed in Stata statistical software release 17 (StataCorp). Two-tailed *P* < .05 was considered statistically significant.

## Results

**Figure 1.**
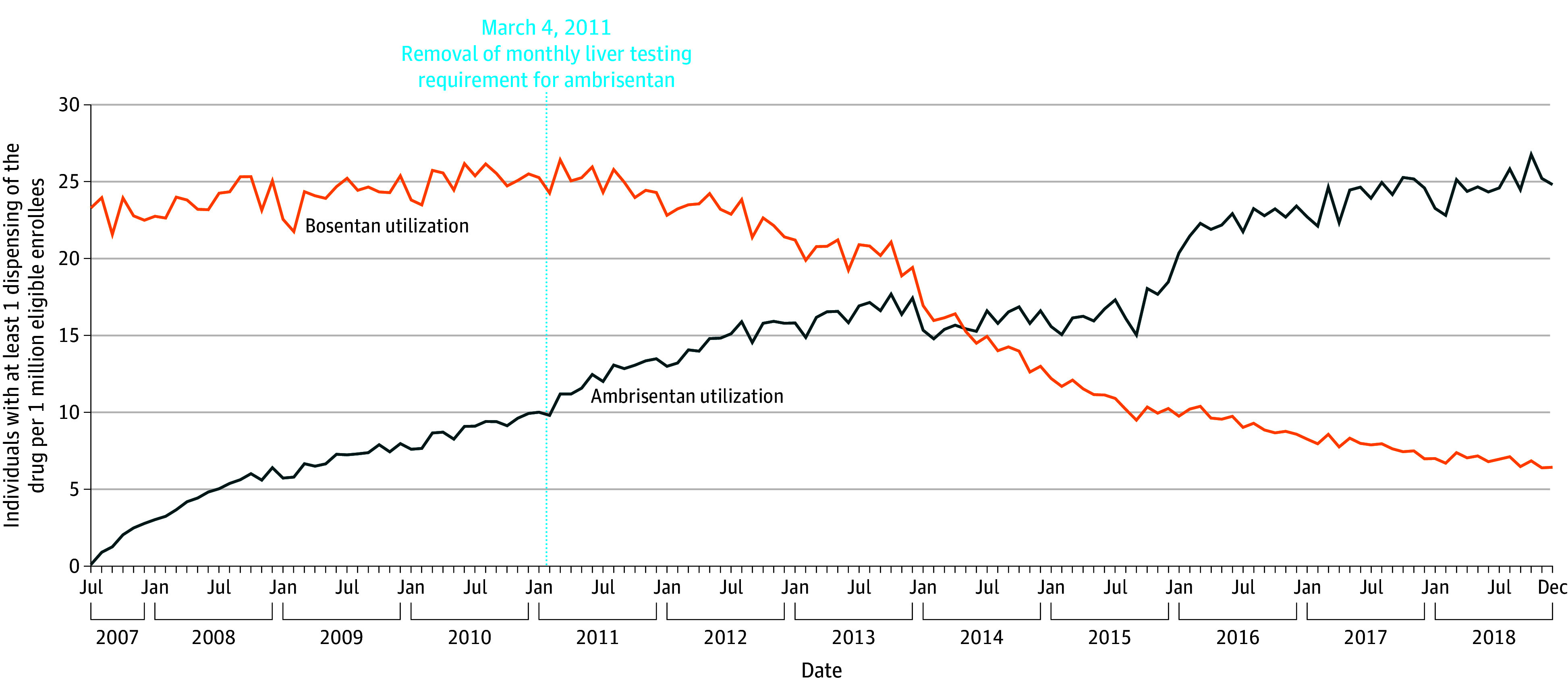
Monthly Use of Ambrisentan and Bosentan, 2007-2018 Until March 4, 2011, the Food and Drug Administration (FDA) required that prescribers of ambrisentan and bosentan obtain liver function tests (LFTs) before initiation of the drug and then every month thereafter while a patient was receiving therapy. The FDA removed this requirement along with the boxed warning for ambrisentan on March 4, 2011, but retained the LFT requirement and boxed warning for bosentan.

A total of 10 261 patients received at least 1 prescription for ambrisentan during the study period (7442 women [72.5%]; mean [SD] age at first fill, 52.6 [17.6] years), and 11 159 patients received at least 1 prescription for bosentan (7931 women [71.1%]; mean [SD] age at first fill, 47.7 [23.7] years). A median (IQR) of 15.4 (8.2-21.2) patients per million enrollees filled prescriptions for ambrisentan each month, and 21.0 (9.8-24.2) patients per million enrollees filled prescriptions for bosentan. Prescriptions for ambrisentan increased during the study period from 0.1 users per million in July 2007 to 24.8 users per million in December 2018. By contrast, prescriptions for bosentan, although stable from July 2007 to early 2012 (ranging from 21.6 to 26.2 users per million), decreased to 6.4 users per million by December 2018 (Figure 1).

### Changes in Dispensing of Ambrisentan vs Bosentan

**Figure 2.**
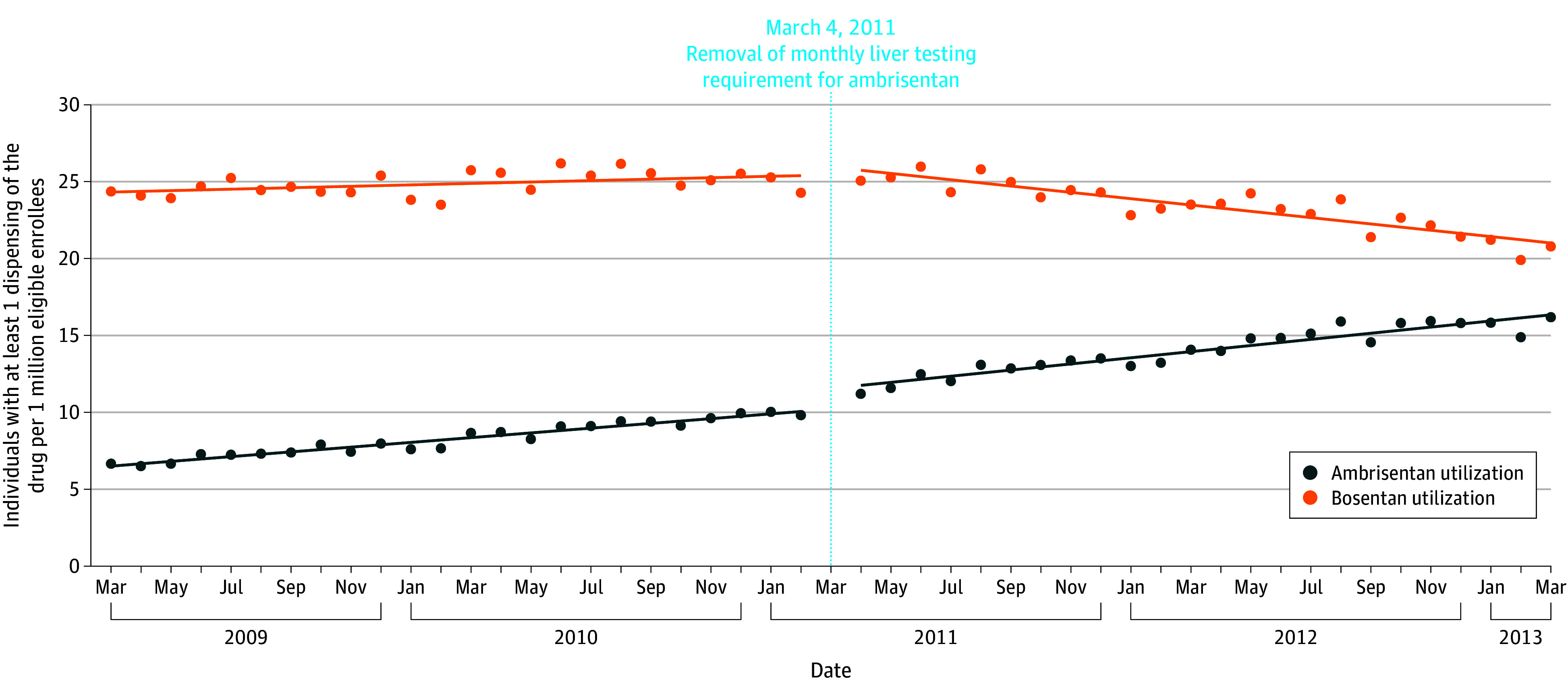
Time Series Analysis of Ambrisentan and Bosentan Use, 2009-2013 Drug use was measured for each month of the study period for individuals enrolled in their insurance plan for at least 28 days that month. On March 4, 2011, the Food and Drug Administration removed the boxed warning and liver function testing requirement for ambrisentan but retained it for bosentan.

In the 2 years leading up to the removal of the boxed warning and LFT requirements for ambrisentan (March 2009 to February 2011), the use of ambrisentan and bosentan increased. Dispensing of ambrisentan increased by a mean of 0.15 patients per million per month (95% CI, 0.14 to 0.17 patients per million per month), whereas dispensing of bosentan increased by 0.05 patients per million per month (95% CI, 0.01 to 0.08 patients per million per month). Removal of the boxed warning for hepatotoxicity and REMS LFT requirement on ambrisentan was associated with an immediate increase in the use of ambrisentan (1.50 patients per million; 95% CI, 1.08 to 1.92 patients per million per month) but no significant change in the use of bosentan (0.55 patients per million; 95% CI, −0.22 to 1.32 patients per million per month). In addition, the rate of use for ambrisentan further increased during the postintervention period (an additional monthly increase of 0.05 patients per million per month above the baseline trend; 95% CI, 0.01 to 0.08 patients per million per month), whereas the use of bosentan decreased (monthly decrease of −0.25 patients per million per month; 95% CI, −0.30 to −0.20 patients per million per month), compared with the monthly baseline trends (Table 1 and Figure 2). The sensitivity analysis excluding the 3-month window (March 2011 to May 2011) immediately following removal of ambrisentan’s liver toxicity warning and LFT requirement yielded similar findings (eTable 2 in Supplement 1).

**Table 1.** Interrupted Time Series Analysis Comparing the Use of Ambrisentan to Bosentan

Measure	Ambrisentan		Bosentan	
	Estimate (95% CI)^a^	*P* value	Estimate (95% CI)^a^	*P* value
Intercept	6.35 (6.17 to 6.53)	<.001	24.27 (23.86 to 24.69)	<.001
Baseline trend	0.15 (0.14 to 0.17)	<.001	0.05 (0.01 to 0.08)	.01
Level change after removal of the LFT requirement for ambrisentan	1.50 (1.08 to 1.92)	<.001	0.55 (−0.22 to 1.32)	.16
Trend change after removal of the LFT requirement for ambrisentan	0.05 (0.01 to 0.08)	.01	−0.25 (−0.30 to −0.20)	<.001

Abbreviation: LFT, liver function test.

^a^ This represents the monthly number of patients with at least 1 dispensing per million enrollees.

### LFT Monitoring Before Initiation of Ambrisentan vs Bosentan

In the years leading up to removal of the boxed hepatotoxicity warning and REMS LFT requirement for ambrisentan (July 2007 to December 2010), observed LFT monitoring in the 90 days before initial dispensing was higher in patients receiving ambrisentan (median [IQR], 77.2% [75.6% to 79.5%]) than in patients receiving bosentan (median [IQR], 71.7% [68.1% to 73.9%]). There was a slight increase in LFT monitoring before initiation of ambrisentan (an absolute increase of 1.1% per month; 95% CI, 0.3% to 1.9% per month) during the baseline period but no significant change in LFT monitoring before initiation of bosentan (0.64% per month increase; 95% CI, −0.2% to 1.5% per month increase).

**Figure 3.**
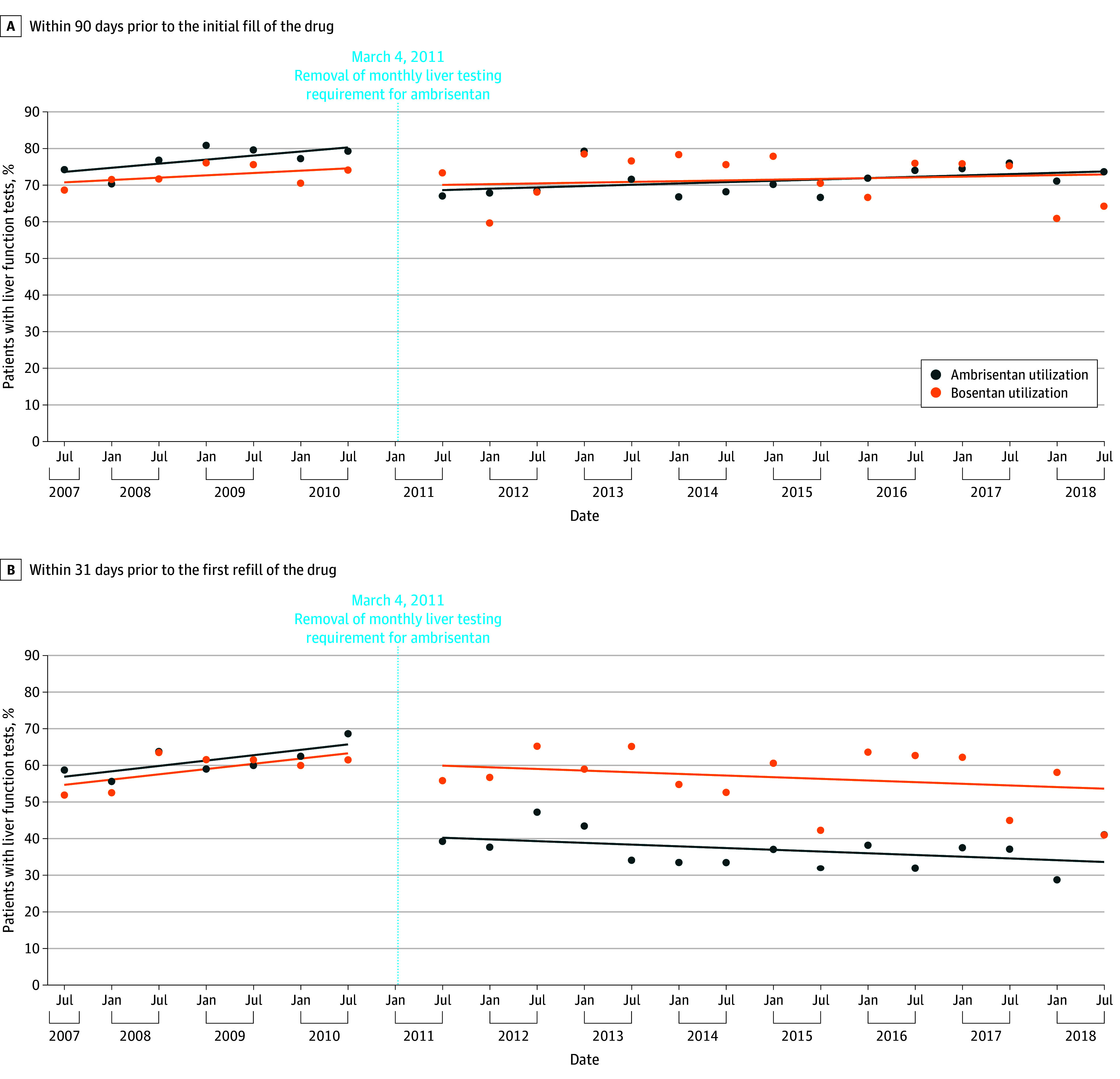
Drug Use Measured for Each Month of the Study Period as the Number of Individuals With at Least 1 Dispensing per 1 000 000 Individuals Enrolled in Their Insurance Plan for at Least 28 Days That Month On March 4, 2011, the Food and Drug Administration removed the boxed warning and liver function testing requirement for ambrisentan but retained it for bosentan.

Removal of the boxed hepatotoxicity warning and the REMS LFT requirement for ambrisentan was associated with a decrease in LFT monitoring before initiation of ambrisentan (−13.1%absolute reduction; 95% CI, −18.2% to −8.0%absolute reduction) but no significant change in LFT monitoring before initiation of bosentan (5.4% reduction; 95% CI, −16.3% to 5.5% reduction). There was no difference in the rate of change in LFT monitoring compared with baseline trends for either group after the boxed warning for hepatotoxicity and REMS LFT requirement for ambrisentan were lifted (Table 2 and Figure 3A). In the postintervention period (July 2011 to July 2018), observed LFT monitoring before the initial dispensing was similar between the ambrisentan group (median [IQR], 71.1% [68.1% to 73.9%]) and the bosentan group (median [IQR], 75.3% [67.4% to 76.3%]).

**Table 2.** Interrupted Time Series Analysis Comparing the Receipt of LFTs Among Patients Filling Ambrisentan vs Bosentan Prescriptions

Measure	Ambrisentan		Bosentan	
	Estimate (95% CI)^a^	*P* value	Estimate (95% CI)^a^	*P* value
LFTs before initial dispensing				
Intercept	72.50 (67.96 to 77.03)	<.001	70.12 (66.99 to 73.24)	<.001
Baseline trend	1.11 (0.30 to 1.92)	.01	0.64 (−0.17 to 1.45)	.12
Level change after removal of the LFT requirement for ambrisentan	−13.10 (−18.21 to −7.99)	<.001	−5.38 (−16.30 to 5.53)	.31
Trend change after removal of the LFT requirement for ambrisentan	−0.75 (−1.64 to 0.14)	.10	−0.43 (−1.83 to 0.96)	.52
LFTs before first refill				
Intercept	55.43 (50.41 to 60.45)	<.001	53.25 (46.37 to 60.13)	<.001
Baseline trend	1.47 (0.35 to 2.59)	.01	1.43 (0.23 to 2.63)	.02
Level change after removal of the LFT requirement for ambrisentan	−26.43 (−34.38 to −18.48)	<.001	−4.33 (−11.4 to 2.75)	.22
Trend change after removal of the LFT requirement for ambrisentan	−1.94 (−3.25 to −0.63)	.01	−1.88 (−3.36 to −0.41)	.02

Abbreviation: LFT, liver function tests.

^a^ For LFTs before the initial dispensing, this represents the percentage of individuals initiating the drug who had LFTs checked within 90 days before drug initiation. For LFTs before first refills, this represents the percentage of individuals refilling their medication who had LFTs checked within 31 days before the refill.

### LFT Monitoring Before Refills of Ambrisentan vs Bosentan

Similar rates of LFT monitoring were observed before patients’ first refills of ambrisentan (median [IQR], 60.0% [58.9% to 63.2%]) and first refills of bosentan (median [IQR], 61.5% [56.3% to 61.6%]) in the baseline period. Removal of the boxed warning for hepatotoxicity and REMS LFT requirement was associated with a 26.4% absolute decrease (95% CI, −34.4% to −18.5% absolute decrease) in LFTs before the first refill of ambrisentan but no significant change in LFTs before the first refill of bosentan. In the postintervention period, both groups saw a significant decrease in the percentage of patients undergoing LFTs before their first refill, compared with baseline trends (Table 2 and Figure 3B). The observed percentage of patients getting LFTs checked before their first refill in the postintervention period was lower for those taking ambrisentan (median [IQR], 37.1% [33.5% to 38.8%]) than those taking bosentan (median [IQR], 58.1% [53.7% to 62.5%]).

## Discussion

This serial cross-sectional study found that the removal of the liver toxicity warning and REMS LFT monitoring requirement for ambrisentan was associated with increased use of the drug and reduced laboratory monitoring in the years after removal. No such changes were observed for bosentan, which has the same indications as ambrisentan but for which the liver toxicity warning and REMS LFT monitoring requirement remained in place throughout the study period. The impact of removing ambrisentan’s LFT requirement was more pronounced for testing before the first refill of the drug compared with testing before drug initiation. During the postintervention period, just over one-third of ambrisentan initiators had LFTs checked before their first refill of the drug, whereas two-thirds had LFTs checked before their initial dispensing. Our findings underscore how labeling changes and removal of a REMS-related requirement can lead to shifts in prescribing and testing behavior, in this case among physicians managing PAH.

At least 2 separate hypotheses could explain the increased use of ambrisentan and decreased laboratory monitoring following March 2011. First, the new safety data underpinning the decision may have convinced clinicians to prescribe the drug more and check LFTs less often. Alternatively, the LFT requirement—and the accompanying time and clinic resources—may have been an impediment to prescribing; without the REMS mandate for laboratory testing, physicians may have been more likely to prescribe the drug and less likely to check LFTs. Disentangling these hypotheses is beyond the scope of this article, although we have begun follow-up work interviewing PAH prescribers to better understand their motivations for prescribing and laboratory monitoring in the REMS programs.^11^

Although LFT monitoring declined after the FDA eased the REMS on ambrisentan, physicians continued to check LFTs at fairly high rates in the postintervention period, particularly before initial dispensing of the drug. The median percentage of patients who had LFTs checked in the postintervention period before their initial dispensing of ambrisentan was only 4% less than for bosentan (71.1% vs 75.3%) despite the minimal risks of ambrisentan hepatotoxicity. Persistent LFT monitoring for patients with ambrisentan could be a function of clinical inertia or risk aversion among prescribers accustomed to checking LFTs in patients taking endothelin-receptor antagonists. Further work could examine whether long-standing abrisentan and bosentan prescribers were more likely to continue checking LFTs compared with newer prescribers.

Some patients taking ambrisentan may also have had LFTs checked for other reasons—for example, because they were taking different medications associated with hepatotoxicity or had known liver disease. We did not analyze baseline rates of LFTs in matched cohorts of patients who were not taking these drugs for PAH. Nonetheless, by using bosentan as a control for ambrisentan, we were able to see that LFT monitoring before ambrisentan initiation in the postintervention period was nearly as high as LFT monitoring for bosentan, a drug with well-known hepatotoxicity and a longstanding REMS requirement in place.

The comparable rates of LFT monitoring before initiation of ambrisentan and bosentan, even after the REMS requirement was lifted on ambrisentan, also highlight the suboptimal rates of testing for patients prescribed bosentan. Our findings were similar to those observed in an earlier study of LFTs in users of bosentan (from 2001 to 2013),^18^ and both are substantially less than the 100% anticipated by the implementation of a REMS requirement. Our study captures LFTs that patients actually obtained, not just tests that physicians ordered, and so rates of LFT ordering by physicians may be higher. Our dataset could also be failing to capture a small number of tests that patients received outside of their insurance plans but that were nevertheless visible to prescribers and pharmacists in a way that would satisfy the REMS requirement. To minimize the chances of missing laboratory tests covered by other insurers, all patients in our cohort had at least 180 days of continuous enrollment in the claims databases leading up to and including their first ambrisentan or bosentan fill (and 31 days of continuous enrollment leading up to their first refill), and we assumed that tests were done when patients were hospitalized. Further studies are needed to understand why patients manage to fill prescriptions for the drug seemingly without necessary testing and whether this is a problem facing a subset of prescribers and practices or is more systemic.

### Limitations

A few important limitations of our analysis should be noted. First, although segmented regression analysis controls for underlying trends, it may be confounded by other changes that occurred either at the time of the change or during the postintervention period. However, we are aware of no other major changes related to the use of ambrisentan and bosentan that occurred in 2011. In addition, we tried to control for other changes by limiting the observation period to 2 years preintervention and postintervention in the analysis of drug use, and by analyzing a control group (individuals taking bosentan) who would be subject to the same changes, with the exception of the removal of LFT requirement. Second, the model assumes a linear trend in the outcome within each segment. Third, the limited number of prechange points for LFT monitoring and the decreasing numbers of individuals initiating bosentan may have resulted in less stable estimates and, potentially, lack of statistical power in the evaluation of LFT requirements.

## Conclusions

In this serial cross-sectional study, removal of the boxed hepatotoxicity warning and the REMS LFT requirement were associated with increased use of ambrisentan and decreased LFT monitoring. However, LFT monitoring of ambrisentan remained high during the study period, whereas LFT monitoring of bosentan remained below complete adherence. Further clinician education may be needed to maximize the benefits of REMS programs and labeling warnings designed to protect patients and ensure the safe administration of high-risk medications.

## Notes

Supplement 1. **eTable 1.** Definitions of liver function tests **eTable 2.** Sensitivity analysis for the interrupted time-series comparing the use of ambrisentan to bosentan, excluding March 2011-May 2011 from the analysis^a^

Supplement 2.
Data Sharing Statement
