# Supplementary material for: Removing the FDA’s Boxed Hepatotoxicity Warning and Liver Function Testing Requirement for Ambrisentan
Source: JAMA Netw Open. 2024 Jul 18;7(7):e2419873. doi: 10.1001/jamanetworkopen.2024.19873 (PMC11258588; doi:10.1001/jamanetworkopen.2024.19873)
Supplement: Supplement 1. — eTable 1. Definitions of liver function tests eTable 2. Sensitivity analysis for the interrupted time-series comparing the use of ambrisentan to bosentan, excluding March 2011-May 2011 from the analysisa [file jamanetwopen-e2419873-s001.pdf]

Supplemental Online Content

Feldman WB, Mahesri M, Sarpatwari A, et al. Removing the FDA's boxed hepatotoxicity warning and liver function testing requirement for ambrisentan. *JAMA Netw Open*. 2024;7(7):e2419873. doi:10.1001/jamanetworkopen.2024.19873

eTable 1. Definitions of liver function tests

eTable 2. Sensitivity analysis for the interrupted time-series comparing the use of ambrisentan to bosentan, excluding March 2011-May 2011 from the analysis[a]

This supplemental material has been provided by the authors to give readers additional information about their work.

© 2024 Feldman WB et al. *JAMA Network Open.*

eTable 1: Definitions of liver function tests

CPT codes	Description
84460	TRANSFERASE; ALANINE AMINO
80076	HEPATIC FUNCTION PANEL
80054	COMPREHENSIVE METABOLIC PANEL
80058	HEPATIC FUNCTION PANEL
80053	COMPREHENSIVE METABOLIC PANEL
84450	TRANSFERASE; ASPARTATE AMINO

© 2024 Feldman WB et al. *JAMA Network Open.*

eTable 2: Sensitivity analysis for the interrupted time-series comparing the use of ambrisentan to bosentan, excluding March 2011-May 2011 from the analysis[a]

	Ambrisentan		Bosentan	
**Measure**	**Estimate (95% CI)**	**p-value**	**Estimate (95% CI)**	**p-value**
Intercept	6.35 (6.17 to 6.53)	<0.001	24.27 (23.86 to 24.69)	<0.001
Baseline trend	0.15 (0.14 to 0.17)	<0.001	0.05 (0.01 to 0.08)	0.01
Level change after removal of the LFT requirement	2.08 (1.73 to 2.43)	<0.001	0.26 (-0.52 to 1.04)	0.50
Trend change after removal of the LFT requirement	0.03 (0.01 to 0.06)	0.02	-0.26 (-0.30 to -0.20)	<0.001

LFT: liver function tests

a. This sensitivity analysis excludes the 3 months from March 2011 to May 2011.

© 2024 Feldman WB et al. *JAMA Network Open.*
