# Supplementary material for: Removing the FDA’s Boxed Hepatotoxicity Warning and Liver Function Testing Requirement for Ambrisentan
Source: JAMA Netw Open. 2024 Jul 18;7(7):e2419873. doi: 10.1001/jamanetworkopen.2024.19873 (PMC11258588; doi:10.1001/jamanetworkopen.2024.19873)
Supplement: Supplement 2. — Data Sharing Statement [file jamanetwopen-e2419873-s002.pdf]

Data Sharing Statement

Feldman. Removing the FDA's Boxed Hepatotoxicity Warning and Liver Function Testing Requirement for Ambrisentan. *JAMA Netw Open*. Published July 18, 2024.
doi:10.1001/jamanetworkopen.2024.19873

Data
Data available: Yes
Data types: Other (please specify)
Additional Information: Aggregated claims data
How to access data: wbfeldman@bwh.harvard.edu
When available: With publication

Supporting Documents
Document types: None

Additional Information
Who can access the data: Researchers whose proposed use of the data has been approved.
Types of analyses: For research purposes
Mechanisms of data availability: With a signed data access agreement
